# Association between pan-immune-inflammation value and dyslipidemia in the United States population

**DOI:** 10.3389/fendo.2025.1518304

**Published:** 2025-03-17

**Authors:** Yu Yan, Shanshan Jia, Xingwei Huo, Lu Liu, Lirong Sun, Shuangliang Ma, Xiaoping Chen

**Affiliations:** ^1^ Department of Cardiology, West China Hospital, Sichuan University, Chengdu, Sichuan, China; ^2^ Department of Cardiology, Peking University First Hospital, Beijing, China; ^3^ Second Department of Internal Medicine, Affiliated Hospital of Tibet University for Nationalities, Xianyang, Shaanxi, China

**Keywords:** pan-immune-inflammation value, dyslipidemia, NHANES, inflammation, cross-sectional study

## Abstract

**Object:**

To investigate the possible association between pan-immune-inflammation value (PIV) and dyslipidemia.

**Methods:**

This cross-sectional study used the data obtained from National Health and Nutrition Examination Survey (NHANES). The independent variable used the logarithmic form of PIV-log2 (PIV). The definition of dyslipidemia was based on the National Cholesterol Education Program standards. Weighted multivariate logistic regression analyses, the restricted cubic spline (RCS) and threshold effect analysis were explore the association between PIV and dyslipidemia. Stratified analyses were used to identify potential associations with other covariates. The receiver operating characteristic (ROC) curve was constructed compared to systemic immune-inflammation index (SII).

**Results:**

6,821 participants were included, of whom 47% were male and 77% had dyslipidemia. After adjusting for all confounders, PIV and dyslipidemia had an significantly positive association (OR (95%CI): 1.13 (1.01-1.25); *P =* 0.03). Compared to participants with lowest quartile (Q1) of PIV, participants with the highest quartile (Q4) had a significantly higher risk of dyslipidemia (OR (95%CI): 1.37 (1.05-1.80); *P =* 0.022). The RCS curve showed an inverted J-shaped relationship between PIV and dyslipidemia (*P*-nonlinear = 0.0415, *P*-overall < 0.001). The threshold effect analysis revealed that the inflection point was 9.192. Stratified analyses showed that age and BMI modified the PIV-dyslipidemia relationship (*P* for interaction < 0.05). The ROC curve found that compared with SII, PIV had a similar predictive value (area under curve (AUC): 0.566 vs 0.558; *P* = 0.073).

**Conclusion:**

This study discovered that PIV had a significantly positive relationship with dyslipidemia, especially in young and overweight individuals.

## 1 Introduction

Hyperlipidemia was a condition that was characterized by abnormally elevated blood lipids broadly including increase in low-density lipoprotein cholesterol (LDL-C) and triglycerides (TG) as well as reduce in high-density lipoprotein cholesterol (HDL-C) (1), which could be attributed to a variety of genetic predispositions or acquired health conditions (2). Hereditary hyperlipidemia referred to congenital disorders such as familial hypercholesterolemia, whereas primary hyperlipidemia generally referred to elevations of all lipoproteins (3) (except HDL-C). In adults, hyperlipidemia was recognized to contribute to the development of cardiovascular disease (CVD) (2). The available evidence suggested an association between hyperlipidemia which was characterized by the increase of TG and LDL-C in plasma, and the prevalence of coronary artery disease (4). Not only did it promote atherosclerosis of blood vessels, hyperlipidemia could also act directly on the heart leading to ischemia-reperfusion injury (5). Moreover, the CVD accounted for the highest percentage of all causes of death among adults in the United States, and the risk of developing CVD in people with hyperlipidemia was about twice than those without dyslipidemia (1).

The link between atherosclerosis and hyperlipidemia, as well as the role of persistent low-grade inflammation and lipid abnormalities, has prompted research into the potential association between elevated lipid levels and inflammatory states (6). The cholesterol has been proved to directly induce inflammation by the activation of the NLRP3 inflammasomes, possibly contributing to the initiation and exacerbation of local and systemic immune inflammatory responses (7).

The pan-immune-inflammation value (PIV) represented a novel biomarker for predicting inflammatory status, including four cell types in peripheral blood (8). The definition of it relied on the counts of neutrophils, monocytes, lymphocytes, and platelets (9). In 2020, it was initially used in metastatic colorectal cancer patients, showing a good ability to assess the prognosis of them and it even exceeded that of previously established markers related to inflammation (10). PIV was a potent and independent predictor of coronary slow flow, superior to other inflammatory markers (11). In a cross-sectional study, elevated PIV was associated with an increased risk of all-cause mortality (1.37 (1.20-1.55); *P* < 0.001) and cardiovascular mortality (1.62 (1.22-2.15); *P* < 0.001) in hypertensive patients (12). The systemic immune-inflammation index (SII: platelet count × neutrophil count/lymphocyte count) was applied to the assessment of the risk of dyslipidemia previously, but the correlation between them was relatively small (Model 2: OR (95%CI): 1.03 (1.01-1.05); Model 3: OR (95%CI): 1.02 (1.00-1.04)) (13).

The association between novel composite biomarker PIV and dyslipidemia has not been reported. Therefore, the study is aimed to explore the association between PIV and dyslipidemia and answer whether PIV can be used to assess the risk of dyslipidemia more effectively.

## 2 Methods

### 2.1 Data source and participants

The National Health and Nutrition Examination Survey (NHANES), administered by the National Center for Health Statistics (NCHS), represents a biennial nationwide survey dedicated to examining the health and nutritional status of citizens in the United States. The purpose of this program is to understand as fully as possible the contemporary disease patterns and better help the development of public health services and the optimization of public health policies. The NHANES data is open to the public and able to be freely downloaded via the official website: https://www.cdc.gov/nchs/nhanes/index.htm (14).

**Figure 1:**
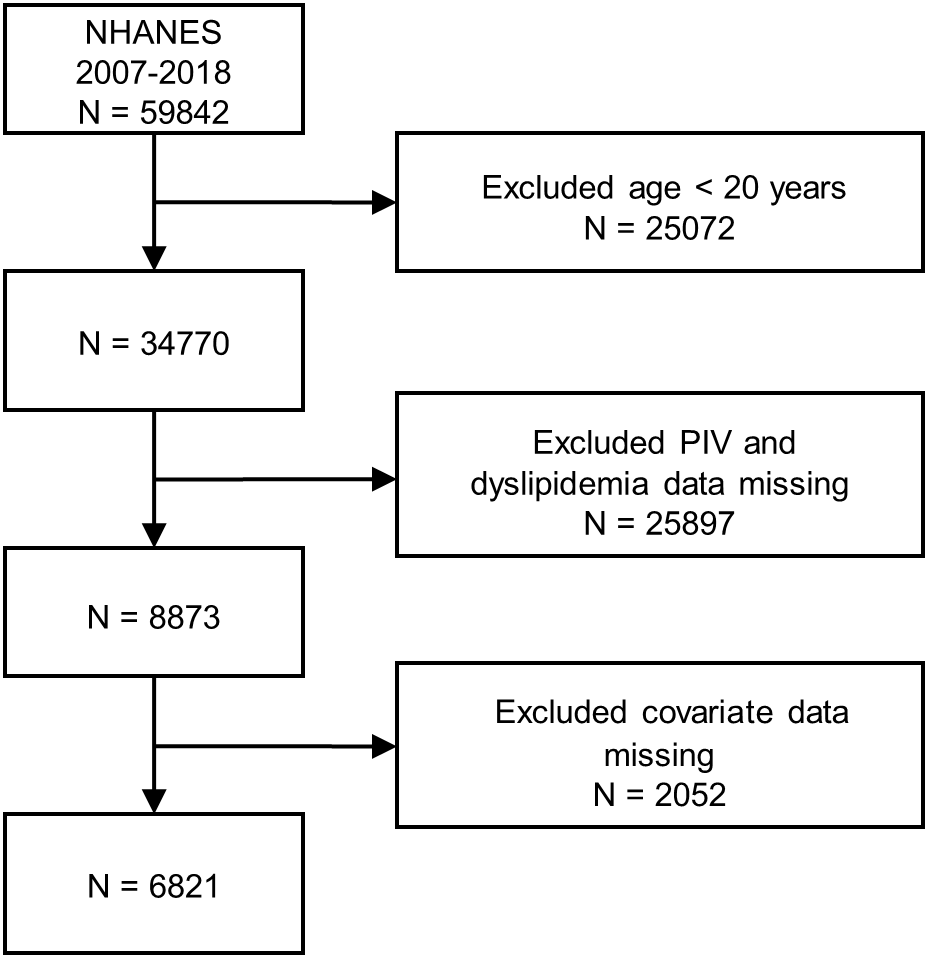
Participant flowchart.

The authors initially included statistics of 59,842 participants of the NHANES (2007–2018) in this study. Data screening for inclusion and exclusion followed the prescribed procedures: (1) participants whose age < 20 years old (N = 25,072); (2) participants without PIV and dyslipidemia data (N = 25,897); (3) participants who had missing covariate data (N = 2,052). A total of 6,821 participants were ultimately selected for subsequent analyses after manual data filtration. The complete flow chart of participant being included and excluded was presented in **Figure 1**.

### 2.2 Assessment of dyslipidemia

Dyslipidemia was defined in accordance with the guidelines developed by the National Cholesterol Education Program Adult Treatment Panel III (NCEP-ATP3). Specifically, dyslipidemia was defined as a total cholesterol (TC) level of 200 mg/dL or higher, TG of 150 mg/dL or higher, HDL-C less than 40 mg/dL for males and 50 mg/dL for females, or LDL-C of 130 mg/dL or higher. Furthermore, subjects who reported taking lipid-lowering drugs were also defined as having dyslipidemia (13).

### 2.3 Assessment of PIV

The formula for calculating PIV was as follows: neutrophil count × platelet count × monocyte count/lymphocyte count. The numeric expression of cell counts was indicated as ×1000 cells/μL (15). Because it was a study based on data of multiple periods, the measurements of target cells in the blood were operated with different instruments in the NHANES mobile examination centers.

### 2.4 Covariates

In order to reduce the influences of confounders on the final results, covariates with known or potential associations with dyslipidemia were selected in the analysis. Demographic variables consisted of gender, race, age, education, body mass index (BMI), poverty-to-income ratio (PIR), smoking, and alcohol use. Hypertension and diabetes were included as medical conditions. Five types of races were divided: Mexican American, non-Hispanic black, non-Hispanic white, other Hispanic and other races. Education levels were classified into three types: less than high school, high school or GED, and above high school. The <25, 25–30, and ≥30 kg/m^2^ BMI degrees were also established. The cut-off points of waist circumference were 102 cm in male individuals and 88 cm in female. The definition of smoking was having smoked 100 or more cigarettes over the lifetime. The definition of alcohol use was the consumption of at least 12 cups of alcoholic beverages in the last 12 months or had a drink of any kind of alcohol over the lifetime. Hypertension was diagnosed as an average SBP ≥ 140 mmHg, or DBP ≥ 90 mmHg based on at least three standard consecutive seated measurements, self-reported hypertension or taking prescription for hypertension. The definition for anti-diabetes consisted of fasting serum glucose ≥ 126 mg/dL, glycated hemoglobin ≥ 6.5%, self-reported diabetes, any diabetic pills or insulin use. Data on creatinine and uric acid were obtained from standard biochemical tests. The history of coronary heart disease, arthritis, stroke, cancer, and congestive heart failure was recorded based on the questionnaires. All respondents were asked to answer “yes” or “no” to corresponding questions.

### 2.5 Statistical analyses

The study took into account the complex, multistage probabilistic sampling design of NHANES and applied appropriate sampling weights. The R software version 4.3.3 (R foundation) was operated for all statistical analyses. In the baseline, weighted means and standard deviations (SD) or interquartile ranges (IQR) were presented for continuous variables, while categorical variables were presented as weighted proportions. The comparative analyses of weighted t-tests and Rao-Scott chi-square tests were carried out to assess the baseline clinical characteristics of each group of patients. The PIV performed a log2 transformation before regression analysis due to its right-skewed nature of the distribution. Weighted logistic regression analyses were carried out to explore the association between PIV and dyslipidemia. The odds ratio (OR) and 95% confidence interval (CI) were calculated for each one-unit increase in PIV as well as for each PIV quartile. The study constructed three regression models: Model 1, adjusted for non-covariates; Model 2, adjusted for age, gender, race, and education levels, PIR; and Model 3, adjusted for all covariates (added BMI, creatinine, uric acid, coronary heart disease, arthritis, stroke, cancer, congestive heart failure, smoking, alcohol use, diabetes and hypertension to Model 2). Additionally, the restricted cubic spline (RCS) and threshold effect analysis were performed to assess the nonlinear relationship and effect size between PIV and dyslipidemia. Stratified analyses for age, gender, BMI, education level, hypertension, diabetes, smoking and alcohol use, were conducted to explore the potential heterogeneity in different subgroups. The receiver operating characteristic (ROC) curve analysis was applied to compare the predictive value of PIV and SII for dyslipidemia. Only bilateral *P*-value of < 0.05 for all statistical tests were considered significant.

## 3 Results

### 3.1 Statistics of baseline

There were all baseline characteristics presented in **Table 1** of 6,821 individuals categorized by whether they had dyslipidemia or not, 47% of whom were male. And there were 77% of participants having dyslipidemia. Except for two variables gender and PIR (*P* > 0.05), all the remaining variables including PIV had significant associations with dyslipidemia (with or without) (*P* < 0.05). The characteristics of people who were susceptible to have dyslipidemia were as follows: older, non-Hispanic white, not above high school, BMI ≥ 25 kg/m^2^, larger waist circumference, higher creatinine and uric acid, hypertensive, diabetic, drinkers, nonsmokers, had the history of coronary heart disease, arthritis, stroke, cancer, congestive heart failure, as well as higher PIV.

**Table 1:** Clinical characteristics of participants by groups whether had dyslipidemia or not.

Characteristic	Dyslipidemia			*P* value
	Overall, N = 6821 (100%)	0, N = 1456 (23%)	1, N = 5365 (77%)	
Age (years, mean ± SE)	51.88 (0.33)	43.65 (0.59)	54.39 (0.31)	<0.001
Gender (%)				0.3
male	3,260 (47%)	711 (49%)	2,549 (47%)	
female	3,561 (53%)	745 (51%)	2,816 (53%)	
Race (%)				<0.001
Mexican American	786 (6%)	147 (6%)	639 (6%)	
Other Hispanic	697 (5%)	116 (5%)	581 (5%)	
Non-Hispanic White	3,037 (71%)	563 (67%)	2,474 (73%)	
Non-Hispanic Black	1,400 (10%)	388 (13%)	1,012 (9%)	
Other Races	901 (7%)	242 (9%)	659 (7%)	
Education level (%)				<0.001
Less than high school	1,315 (12%)	200 (8%)	1,115 (13%)	
High school or GED	1,478 (22%)	271 (18%)	1,207 (23%)	
Above high school	4,028 (66%)	985 (74%)	3,043 (64%)	
PIR (median[IQR])	3.30 (1.74, 5.00)	3.37 (1.69, 5.00)	3.26 (1.75, 5.00)	0.9
BMI (kg/m^2^, %)				<0.001
< 25	1,746 (26%)	570 (39%)	1,176 (22%)	
≥ 25 and < 30	2,286 (34%)	441 (32%)	1,845 (34%)	
≥ 30	2,789 (41%)	445 (29%)	2,344 (44%)	
Waist circumference (cm, mean ± SE)	101.52 (0.34)	95.49 (0.69)	103.36 (0.36)	<0.001
Neutrophil (1000 cells/μl, mean ± SE)	3.97 (0.03)	3.65 (0.06)	4.07 (0.04)	<0.001
Platelet (1000 cells/μl, mean ± SE)	236.34 (1.21)	227.30 (2.00)	239.10 (1.27)	<0.001
Monocyte (1000 cells/μl, mean ± SE)	0.54 (0.00)	0.52 (0.01)	0.55 (0.01)	<0.001
Lymphocyte (1000 cells/μl, mean ± SE)	1.99 (0.02)	1.92 (0.02)	2.01 (0.02)	<0.001
Total cholesterol (mg/dL, mean ± SE)	193.98 (0.87)	167.81 (0.73)	201.97 (0.92)	<0.001
Triglyceride (mg/dL, mean ± SE)	119.14 (1.31)	72.04 (1.01)	133.52 (1.36)	<0.001
LDL-cholesterol (mg/dL, mean ± SE)	115.50 (0.68)	94.12 (0.72)	122.03 (0.75)	<0.001
HDL-cholesterol (mg/dL, mean ± SE)	54.65 (0.32)	59.28 (0.59)	53.23 (0.35)	<0.001
Creatinine (μmol/L, mean ± SE)	78.41 (0.45)	76.24 (0.73)	79.07 (0.53)	0.002
Uric acid (umol/L, mean ± SE)	327.94 (1.36)	308.57 (2.92)	333.86 (1.59)	<0.001
Cholesterol-lowering drugs				<0.001
Yes	2,842 (37%)	0 (0%)	2,842 (48%)	
No	3,979 (63%)	1,456 (100%)	2,523 (52%)	
Hypertension (%)				<0.001
No	3,307 (54%)	997 (73%)	2,310 (48%)	
Yes	3,514 (46%)	459 (27%)	3,055 (52%)	
Diabetes (%)				<0.001
No	5,200 (82%)	1,282 (92%)	3,918 (79%)	
Yes	1,621 (18%)	174 (8%)	1,447 (21%)	
Alcohol use (%)				0.042
No	5,165 (81%)	1,125 (83%)	4,040 (80%)	
Yes	1,656 (19%)	331 (17%)	1,325 (20%)	
Smoking (%)				<0.001
No	3,095 (45%)	567 (39%)	2,528 (47%)	
Yes	3,726 (55%)	889 (61%)	2,837 (53%)	
Coronary heart disease (%)				<0.001
Yes	383 (5%)	17 (1%)	366 (6%)	
No	6,438 (95%)	1,439 (99%)	4,999 (94%)	
Arthritis (%)				<0.001
Yes	2,326 (32%)	310 (19%)	2,016 (36%)	
No	4,495 (68%)	1,146 (81%)	3,349 (64%)	
Stroke (%)				<0.001
Yes	322 (4%)	32 (1%)	290 (4%)	
No	6,499 (96%)	1,424 (99%)	5,075 (96%)	
Cancer (%)				<0.001
Yes	806 (12%)	107 (7%)	699 (14%)	
No	6,015 (88%)	1,349 (93%)	4,666 (86%)	
Congestive heart failure (%)				<0.001
Yes	267 (3%)	19 (1%)	248 (4%)	
No	6,554 (97%)	1,437 (99%)	5,117 (96%)	
PIV (median[IQR])	228.85 (151.01, 357.00)	191.24 (132.16, 305.21)	239.77 (157.25, 372.65)	<0.001
Log2piv (mean ± SE)	7.85 (0.02)	7.65 (0.04)	7.91 (0.02)	<0.001

Baseline data shown above were weighted by Fasting Subsample 2 Year MEC Weight/6.

PIR, poverty-to-income ratio; BMI, body mass index; SBP, systolic blood pressure; DBP, diastolic blood pressure; PIV, pan-immune-inflammation value.

### 3.2 Association between PIV and dyslipidemia

**Figure 2:**
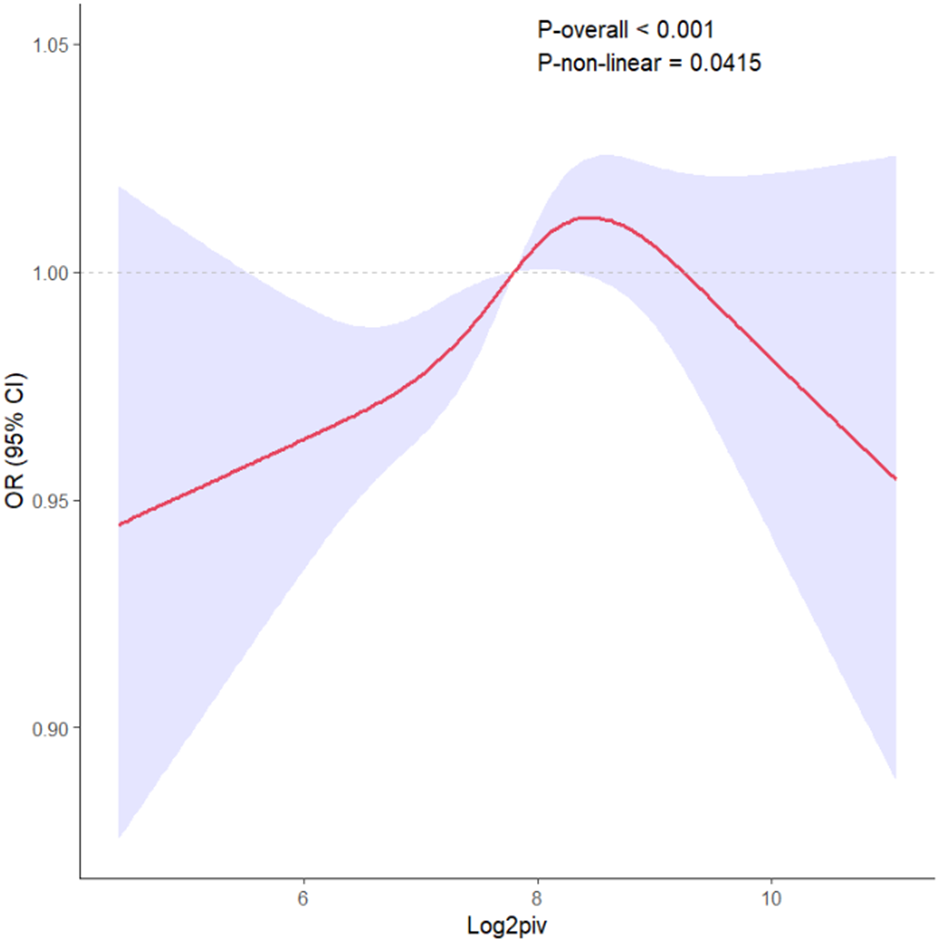
The RCS curve of the association between PIV and dyslipidemia odds ratio in the NHANES 2007–2018. RCS regression was adjusted for age, gender, race, education levels, PIR, BMI, waist circumference, creatinine, uric acid, coronary heart disease, arthritis, stroke, cancer, congestive heart failure, smoking, alcohol use, diabetes and hypertension. RCS, restricted cubic spline; PIV, pan-immune-inflammation value.

All results of the multivariable logistic regression analyses between PIV and dyslipidemia were presented in **Table 2**. The associations in three models were all statistically significant: model 1 (1.33 (1.21-1.47)), model 2 (1.25 (1.12-1.39)) and model 3 (1.13 (1.01-1.25)). In addition, when the independent variable-PIV was divided into four quartiles, the multivariable logistic regression analysis adjusted for all covariates revealed that the ORs for the risk of dyslipidemia compared to Q1 were 1.16 (*P* = 0.2), 1.49 (*P <* 0.001), and 1.37(*P* = 0.022) respectively. Meanwhile, the RCS curve (included all variables adjusted in model 3) displayed an inverted J-shaped relationship of PIV with dyslipidemia (*P*-nonlinear = 0.0415, *P*-overall < 0.001) in **Figure 2**. The threshold effect analysis in **Table 3** revealed that before the inflection point of 9.192, the odds of presenting dyslipidemia increased by 0.127 (*P* < 0.001) with each unit increase in PIV. However, beyond the inflection point, each unit increase in PIV was associated with the decreased risk of dyslipidemia (OR (95% CI): 0.678 (0.475-0.991); *P* = 0.037).

**Table 2:** Association between PIV and dyslipidemia.

PIV	Dyslipidemia OR (95%CI), *P*-value		
	Model 1	Model 2	Model 3
Log2piv	1.33 (1.21, 1.47) <0.001	1.25 (1.12, 1.39) <0.001	1.13 (1.01, 1.25) 0.03
Log2piv Tertile			
Q1	Reference	Reference	Reference
Q2	1.24 (0.99, 1.55) 0.06	1.24 (0.99, 1.55) 0.061	1.16 (0.92, 1.47) 0.2
Q3	1.83 (1.51, 2.22) <0.001	1.71 (1.39, 2.12) <0.001	1.49 (1.21, 1.85) <0.001
Q4	2.13 (1.66, 2.73) <0.001	1.78 (1.36, 2.32) <0.001	1.37 (1.05, 1.80) 0.022
*P* for trend	<0.001	<0.001	0.002

Data are presented as OR (95%CI). OR, odds ratio; CI, confidence interval; PIV, pan-immune-inflammation value; Q1, 1st quartile; Q2, 2nd quartile; Q3, 3rd quartile; Q4, 4th quartile.

Model 1: adjusted for non-covariates.

Model 2: adjusted for adjusted for age, gender, race, education levels, PIR.

Model 3: further adjusted for BMI, waist circumference, creatinine, uric acid, coronary heart disease, arthritis, stroke, cancer, congestive heart failure, smoking, alcohol use, medical conditions (diabetes and hypertension).

**Table 3:** Threshold effect analysis of the relationship between PIV and dyslipidemia.

Results of threshold effect analysis	Outcome	*P* value
Model 1	–	–
Standard linear regression	1.083 (1.015-1.157)	0.017
Model 2	–	–
Break point	9.192	–
<9.192	1.127 (1.048-1.212)	0.001
>9.192	0.678 (0.475-0.991)	0.037
P for likelihood ratio test	–	0.015

### 3.3 Stratified analysis

In order to investigate whether the relationship between PIV and dyslipidemia was heterogeneous among all subgroups, stratified analyses were conducted within fully adjusted models. Most of the interaction tests of the covariates-gender, education level, waist circumference, smoking, alcohol use, hypertension and diabetes were not meaningful (*P* for interaction > 0.05), except for age and BMI. All stratified analysis results were shown in **Table 4**.

**Table 4:** Stratified analysis for the association between PIV and dyslipidemia.

Subgroup	OR (95%)	*P* value	*P* for interaction
Age			0.008
20-39	1.31 (1.12, 1.52)	<0.001	
40-59	1.02 (0.87, 1.19)	0.6	
60~	1.12 (0.96, 1.31)	0.15	
Gender			>0.9
Male	1.19 (1.01, 1.40)	0.037	
Female	1.11 (0.96, 1.29)	0.2	
Education level			0.2
Under high school	1.18 (0.97, 1.45)	0.1	
High school / GED	0.94 (0.75, 1.17)	0.6	
Above high school	1.19 (1.07, 1.34)	0.002	
BMI			0.046
<25	1.11 (0.93, 1.32)	0.3	
≥25 and <30	1.31 (1.14, 1.51)	<0.001	
≥30	1.00 (0.85, 1.18)	>0.9	
Waist circumference			0.3
**≤**102(M) or ≤88(F)	1.15 (1.02, 1.31)	0.025	
>102(M) or >88(F)	1.11 (0.97, 1.27)	0.12	
Hypertension			0.5
No	1.16 (1.03, 1.32)	0.013	
Yes	1.09 (0.94, 1.26)	0.2	
Diabetes			0.2
No	1.15 (1.02, 1.29)	0.017	
Yes	1.03 (0.83, 1.29)	0.8	
Alcohol use			0.8
No	1.13 (0.90, 1.41)	0.3	
Yes	1.14 (1.00, 1.29)	0.042	
Smoking			0.7
No	1.15 (1.01, 1.31)	0.033	
Yes	1.12 (0.96, 1.31)	0.13	

BMI, body mass index; PIR, poverty-to-income ratio; PIV, pan-immune-inflammation value.

### 3.4 ROC analysis

**Supplementary Table 1** and **Supplementary Figure 1** showed the comparison of predictive value of PIV and SII for dyslipidemia. The area under curve (AUC) of PIV (0.566 (0.550, 0.583)) and SII (0.558 (0.542, 0.575)) demonstrated that there was no significance in predictive value between the two indexes (*P* = 0.073).

## 4 Discussion

In the present study, the authors selected 6,821 adults in United States from the NHANES datasets. The important discovers of this cross-sectional study were as follows: (1) PIV index had an significantly positive association with the risk of dyslipidemia. And this effect maintained stable in both continuous and quartile independent variables even after adjustment for all confounders. The prevalence of dyslipidemia was relatively high in older, non-Hispanic white, not above high school, BMI ≥ 25 kg/m^2^, larger waist circumference, higher creatinine and uric acid, hypertensive, diabetic, drinkers, nonsmokers, had the history of coronary heart disease, arthritis, stroke, cancer, congestive heart failure, as well as higher PIV. (2) In the stratified analyses, except for age and BMI, the interaction tests for the rest were not statistically significant (*P* > 0.05). Elevated PIV significantly increased the prevalence of dyslipidemia especially in young (20-39 years old) and overweight (25–30 kg/m^2^) individuals.

To our knowledge, the association between PIV and dyslipidemia was first reported in this study based on a national population of the United States. The PIV was derived from the four important immune cells in plasma, neutrophils, monocytes, lymphocytes, and platelets (15).

The long-term existence of inflammation could cause abnormal lipid metabolism (16). In chronic inflammation, the direction of lipid synthesis altered, manifesting in a reduction of HDL and an elevation in TG levels (17). In individuals with primary Sjogren’s syndrome (an chronic inflammatory autoimmune disease), interleukin-2 was positively correlated with LDL-C (r = 0.7, *P* = 0.02) (18). In addition, interleukin-6 (r = 0.39, *P* = 0.01) and tumor necrosis factor-alpha (TNF-α) had an significant association with TG (r = 0.4, *P* = 0.007) and HDL-C (r = -0.4, *P* < 0.001) (18). Among patients with heterozygous familial hypercholesterolemia, nuclear factor-kappa B (NF-kB) activity of monocytes in blood was independently associated with apolipoprotein B (r = 0.287, *P* = 0.03) and oxidized LDL (r = 0.300, *P* = 0.02) (19). In newly diagnosed patients with metabolic syndrome, TNF-α was significantly associated with fasting blood glucose (r = 0.179, *P* = 0.021), LDL-C (r = 0.199, *P* = 0.01), atherogenic index (r = 0.219, *P* = 0.004), TG (r = 0.351, *P* < 0.001), and HDL-C (r = -0.244, *P* = 0.001) (20). Among individuals without severe cardiovascular risks, there was a positive relationship between serum TG and high-sensitivity C-reactive protein (CRP) (r = 0.298, *P* < 0.001) (21). The relationship between inflammation and cholesterol was complementary in CVD. Excessive migration of LDL to the artery wall triggered an inflammatory cascade, which then accelerated the accumulation of cholesterol, further exacerbating the inflammatory response. This vicious cycle could ultimately lead to the atherosclerosis (22). Modified LDLs had the ability to activate the toll-like receptors, thereby priming the Nod-like receptor protein 3 inflammasomes and ultimately leading to the activation of interleukin-1β and secondary inflammatory responses (23). It was also reported that the lipoprotein-mediated enhancement of inflammation was mainly mediated by TG-rich lipoproteins, not LDL (24). Hypertriglyceridemia enriched with apolipoproteins C-III, could activate toll-like receptor 2 and NF-kB inflammatory signaling pathways, leading to development of atherosclerosis (25). In addition, remnant cholesterol calculated from the equation TC-HDL-LDL was rich in TG (25). In our study, PIV had an inverted J-shaped relationship with TG and remnant cholesterol, but a negative correlation with HDL-C (**Supplementary Figure 2**). There was no significant association between PIV and LDL-C or TC (P > 0.05). *In vivo*, higher TG concentrations were associated with higher levels of plasma glycoproteins which were reliable biomarkers reflecting chronic inflammation (26). Meanwhile, a stronger association of TG or remnant cholesterol with inflammation than LDL-C was similar to our study (27, 28). The inverted J-shaped curve might be explained by the phenomenon (26) that the proportion of TG in lipoprotein began to decrease when it reached a certain degree of inflammatory state, and the increase of other components such as cholesterol and phospholipids was more conducive to the maintenance and further development of inflammation.

In this study, the association between PIV and dyslipidemia was modified by age and BMI. Interestingly, this effect was more significant in younger adults (20-39 years). In a cohort study from China (29), the risk of cardiovascular diseases and all-cause mortality was greatest in people younger than 45 years old with hypertriglyceridemia. Unhealthy lifestyle habits such as smoking, alcohol consumption, high-calorie diets and lack of exercise might be the cause of dyslipidemia in such patients (30). These adverse factors were also associated with intestinal dysbiosis and non-specific inflammation, eventually leading to a chronic low-grade inflammatory state of the body (31). A large cohort study in Dutch (32) revealed that the prevalence of non-LDL dyslipidemia (high triglycerides, high remnant cholesterol and low HDL-C) was highest in men aged 35 to 55. Obesity, smoking and diabetes were important risk factors for this kind of dyslipidemia (32). In addition, chronic inflammation and dyslipidemia were common in patients with higher BMI (33, 34). Elevated levels of interleukin-6 and TNF-α in obese individuals could increase insulin resistance and cardiovascular risk (35). A cohort study from Japan showed that women with BMI ≥ 25 kg/m^2^ had a 1.54-fold higher risk of dyslipidemia than BMI < 25 kg/m^2^ (36). BMI and waist circumference both had a significant positive relationship with dyslipidemia in adults over thirty-five years old in two thousand Chinese (37). The levels of inflammatory markers also increased in obese patients and were positively correlated with BMI (38). In obese, more pro-inflammatory M1 macrophages than anti-inflammatory M2 macrophages existed in adipose tissue (39). The obese mice with hepatocyte -specific PPARα knockout exhibited higher triglycerides in plasma and promoted a shift from M2 to M1 macrophages, thereby activating inflammatory responses such as elevated levels of TNF-α (40). It was also reported that HDL-C could be oxidized by myeloperoxidase in neutrophils and macrophages, hindering cholesterol transport and inducing inflammation (41). Given the increasing prevalence of overweight and obesity in adolescents and young adults (42), future treatment strategies should focus on optimizing lifestyle and weight management to reduce the risks of chronic inflammation and dyslipidemia in this group.

## 5 Strengths and limitations

There were several strengths and limitations in the study. The authors performed statistical analyses using a large, nationally representative sample with adjustment for demographic, examination, questionnaire, and laboratory covariates to ensure that the findings are generalizable to practice. In addition, the study used a novel inflammatory biomarker PIV, which might be reliable and effective in the assessment of inflammation. Lastly, the definitions of independent and dependent variables in the study were based on standardized laboratory tests, which largely ensured the objectivity and accuracy of the data and avoided recall bias. Some limitations were also considered. First, it was a cross-sectional study which had statistical significance between both variables, but could not verify their causal connection. Second, the study only considered the population in United States, so it was unclear whether the findings could be generalized to other races in the world. Third, although this study adjusted the relevant confounders, there might be other unknown confounders.

## 6 Conclusion

The study discovered a significantly positive association between PIV and dyslipidemia in United States adults, especially in young and overweight individuals. Our findings suggested that weight control in early adulthood might be beneficial to maintain lipid homeostasis and prevent the disruption of chronic inflammation. More studies are needed to explore the relationship between inflammation and lipid status in the future.

## Data Availability

Publicly available datasets were analyzed in this study. This data can be found here: https://www.cdc.gov/nchs/nhanes/index.htm.
