# Supplementary material for: Association between pan-immune-inflammation value and dyslipidemia in the United States population
Source: Front Endocrinol (Lausanne). 2025 Mar 17;16:1518304. doi: 10.3389/fendo.2025.1518304 (PMC11955451; doi:10.3389/fendo.2025.1518304)
Supplement: Supplementary file 1 [file DataSheet1.docx]

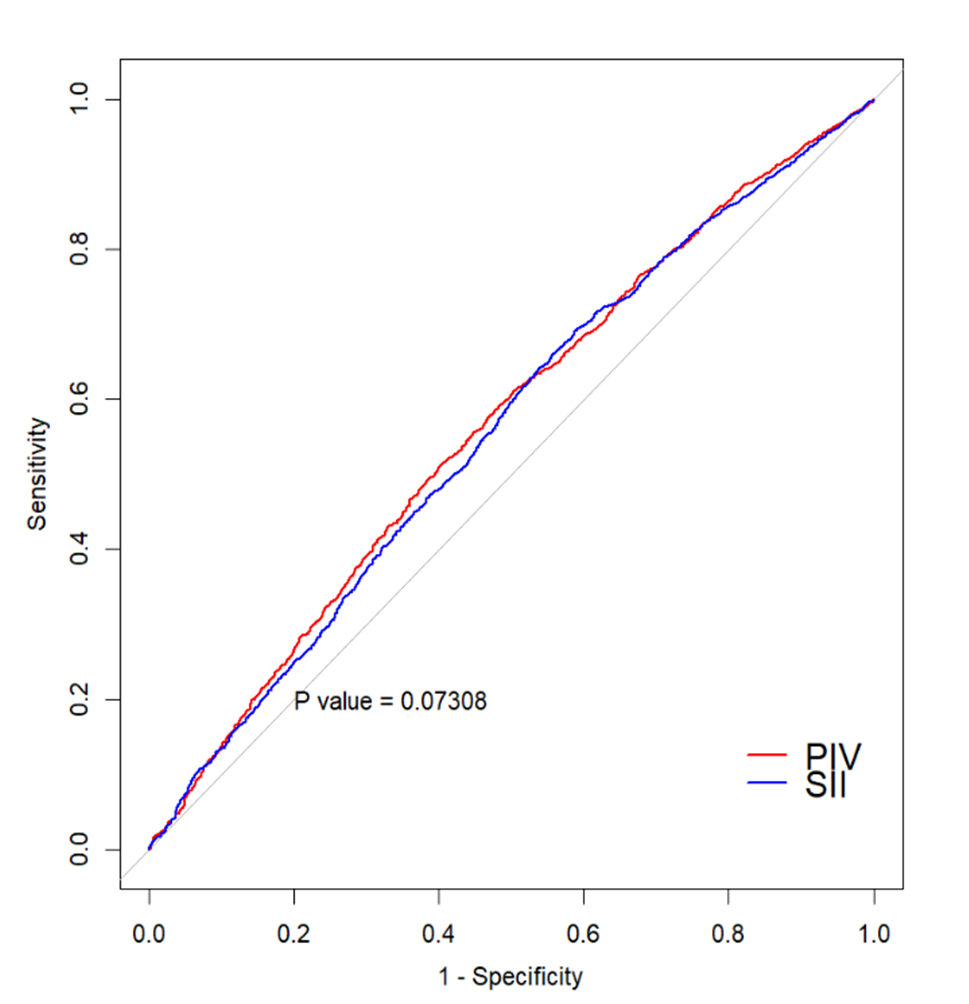


**Supplementary FIGURE 1** ROC analysis. ROC, receiver operating characteristic; PIV, pan-immune-inflammation value; SII, systemic immune-inflammation index.

**
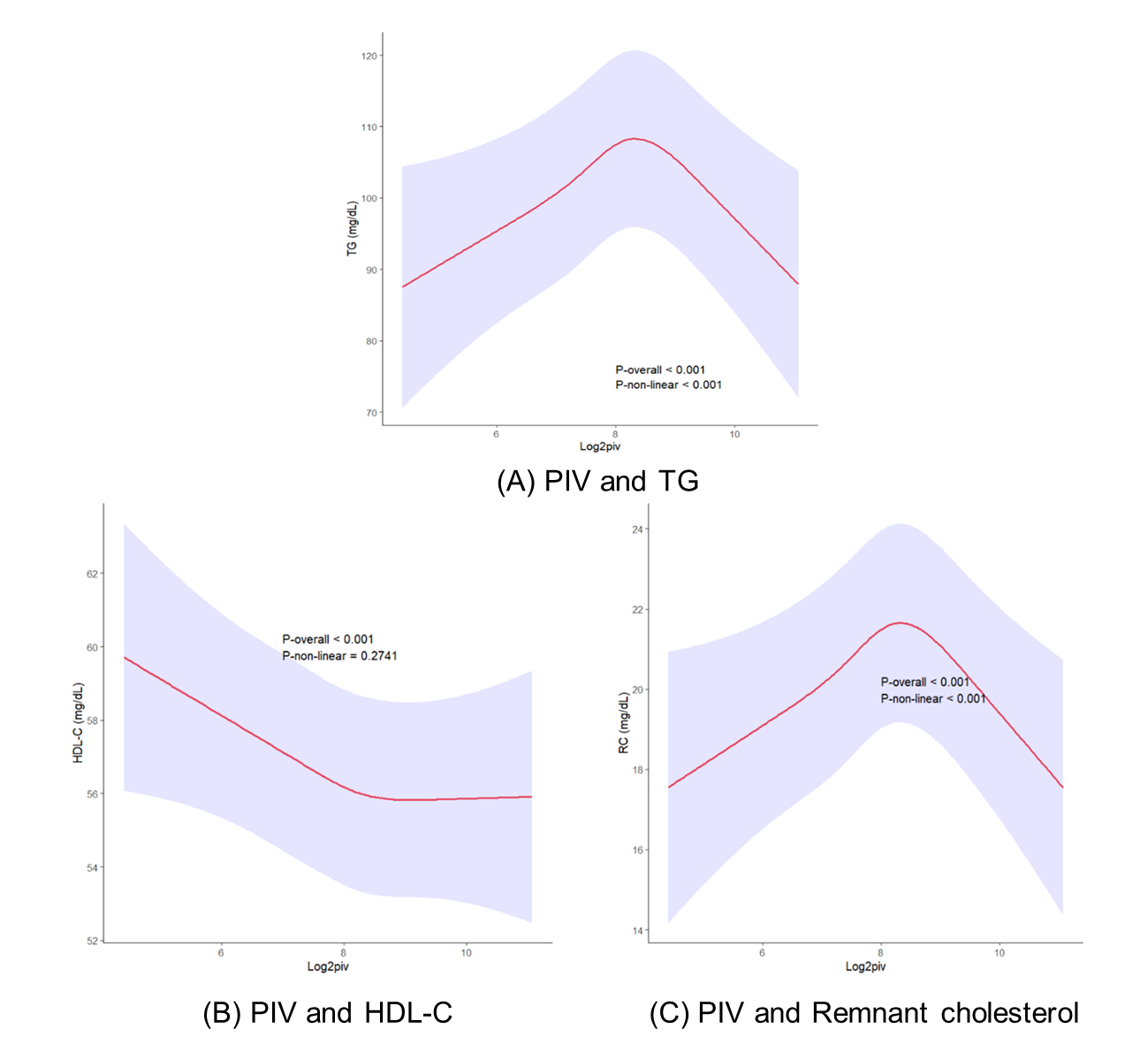
**

**Supplementary FIGURE 2** The RCS curve of the association between PIV and TG (A), HDL (B), Remnant cholesterol (C) in the NHANES 2007–2018. RCS regression was adjusted for age, gender, race, education levels, PIR, BMI, waist circumference, creatinine, uric acid, coronary heart disease, arthritis, stroke, cancer, congestive heart failure, smoking, alcohol use, diabetes and hypertension. RCS, restricted cubic spline; PIV, pan-immune-inflammation value. TG, triglyceride; HDL, high-density lipoprotein cholesterol.
