# Supplementary material for: Association between pan-immune-inflammation value and dyslipidemia in the United States population
Source: Front Endocrinol (Lausanne). 2025 Mar 17;16:1518304. doi: 10.3389/fendo.2025.1518304 (PMC11955451; doi:10.3389/fendo.2025.1518304)
Supplement: Supplementary file 2 [file Table1.docx]

**Supplementary TABLE 1** Comparison of the predictive value of PIV and SII for dyslipidemia based on the ROC curve.

|  | **AUC (95%CI)** | **Cutoff (Sensitivity, Specificity)** | ***P* value** |
| --- | --- | --- | --- |
| PIV | 0.566 (0.550, 0.583) | 7.832 (0.510, 0.400) | 0.07308 |
| SII | 0.558 (0.542, 0.575) | 8.452 (0.693, 0.590) |  |

PIV, pan-immune-inflammation value; SII, systemic immune-inflammation index; ROC, receiver operating characteristic; AUC, area under curve.
